# Observations on the Modus Operandi of Opium

**Published:** 1802-10-01

**Authors:** 

**Affiliations:** Manchester


### *Observations on the Modus Operandi of Opium; communicated by* Mr. WARD, *of Manchester.*

[ Continued from Vol. VII. p. 497 — 506. ]

IN my laſt paper I endeavoured to ſhew, that the *primary* effects reſulting from the *internal* uſe of opium, upon which ſo much ſtreſs has been laid by the advocates for the *ſtimulant doctrine*, are not the conſequence of its operating as a *ſtimulant*, as has been ſuppoſed, but of its operating directly as a ſedative *upon the veſſels and coats of the ſtomach and ſmall inteſtines, and in this manner retarding or interrupting* THE NATURAL FUNCTIONS OF THESE IMPORTANT ORGANS; in other words, that it acts by diminiſhing the motions and powers of motion in the veſſels, *more eſpecially of the arteries*, of the villous and other coats of the ſtomach and bowels; the ſecretory veſſels no longer pour out their accuſtomed quantity of juices; the abſorbents and lacteals, as well as the muſcular coats of the ſtomach, &c. *are thrown into a comparatively quieſcent ſtate*; the cravings of hunger are in conſequence allayed; diſeaſes depending on the natural functions *being carried on with too much rapidity, or which are accompanied with irregular or ſpaſmodic contractions of the muſcular fibres of the alimentary canal*, as vomiting, diarrhœa, teneſmus, &c. are alleviated; *that the reſiſtance which is oppoſed to the free paſſage of the blood through the branches of the arteria ventriculi coronaria, the pylorica, gaſtricæ, and gaſtro epiploicæ, will occaſion a greater determination of blood to the arteria hepatica and ſplenica** *than they can immediately receive*, (in the ſame manner as if a ligature were paſſed round the coronary and other arteries of the ſtomach) *this will cauſe a degree of plethora in the aorta, which will ſoon extend to the heart, the pulmonary, ſubclavian, and carotid arteries, and be the means of increaſing the frequency and ſtrength of their contractions, and the uſual concomitants of an accelerated circulation will enſue*; *and thus, in habits weakened by the exceſſive uſe of opium, an increaſe in the tone and vigour of the ſyſtem is acquired, for a ſhort time*, (which could not have been brought about *by any ſtimulant medicine, internally adminiſtered, becauſe of the tendency which medicines*

* Here we have another ſtrong confirmation of *the ſedative theory* (as well as an opportunity of obſerving its ſimplicity) *in the frequent occurrence of hepatic and ſplenic inflammation, and the rare occurrence of gaſtric inflammation* in thoſe perſons who fall a ſacrifice to the immoderate uſe of opium; and if fermented liquors and ſpirits do not operate in the ſame manner as I am endeavouring to prove with reſpect to opium, how ſhall we explain their ſo conſtantly producing the ſame effects, where taken to exceſs?

*medicines of this clafs have to empty, rather than to fill, the large arteries, by promoting, inftead of retarding, the circulation in the fmaller* feries of arteries both in the vifcera and the extremities) a fenfation of warmth is diffufed through the fyftem,* and the fpirits are raifed fo as to render thofe who were before pale, dull and languid, lively and alert;† but the *delufion* can never be

* Several paffages occur in the Inquiry which feem to imply that opium has the power of augmenting the heat of the body *beyond the healthy ftandard,* "With the increafe of frequency of the pulfe the heat of the body is generally fomewhat augmented." p. 31. "With the increafed frequency of pulfe, the heat of the body is, as abovementioned, fomewhat augmented, at leaft in all my experiments I found this to be the cafe, *if I could judge by my own feelings,* and it was fometimes attended with flufhings of the face." p. 38. "In twenty minutes, my pulfe was fuller as well as quicker; *the heat of my body was raifed.*" p. 66. "Taken internally it quickens and ftrengthens the contractions of the heart and arteries, *increafes the heat of the body in general.*" p. 169, 170.

It appears, however, from the experiments of Dr. Bard (who had recourfe to a more certain teft than the feelings, viz. a thermometer) *that it does not increafe the heat of the body beyond* 98; (indeed, who ever faw the circulation fo much accelerated by opium as to render fuch a circumftance probable?) but it is highly probable that a fenfe of coldnefs muft often, (perhaps always) accompany the languor and depreffion of fpirits which the lovers of opium complain of fo heavily, except when they are under the *primary influence* of their morning or evening dofe; and I have no doubt, was the experiment made, where fo large a dofe has been taken as to produce fuch a degree of languor as is here fuppofed (whether it may have accelerated the circulation *primarily* or not) that the heat would be found reduced much lower than at the commencement of the experiment; the fame may alfo occur in a lefs degree, during that ftate of inactivity which is required in conducting fuch experiments as Dr. Crump was engaged in; and as opium accelerates the circulation primarily, under certain circumftances, as has been explained, and *(when it has that effect) raifes the fpirits (more or lefs in proportion to the degree of languor previoufly fubfifting)* it muft in thefe inftances increafe the heat of the body, *in fome meafure, fo as to bring it nearer to the natural ftandard.*

"Sed ne longius auctores auctoribus opponam, opinionis utriufque veritatem, fequente experimento, detegere conatus fum. Per totum actionis opii ftadium, in plerifque præcedentibus experimentis, thermometrum Fahrenheitiano fimile, fub axilla fubinde conclufi; quo licet per dimidium horæ retentum, nunquam argentum vivum ultra gradum 98um afcendebat, quanquam fani et ab opii potentia immunis, femper ad gradum 98um, et altius fæpe, eodem temporis fpatio naturalis corporis noftri calor, mercurium excitare poteft." Bard, Differtatio Inauguralis de Opio, p. 25, 26.

Whoever will perufe Dr. Bard's Thefis, will find (notwithftanding the hafty manner in which it is paffed over by Dr. Crump, fee Inquiry, p. 36, 37,) that it contains many ingenious and valuable obfervations, equally creditable to the acutenefs and candour of the author.

† Dr. Ruffell remarks in his Hiftory of Aleppo, that the ufe of opium is not fo common among the Turks as is generally imagined, being chiefly confined to debauchees. The immediate effect I obferved it to have, (fays he)

be of long continuance, for in general by the time fifty or ſixty minutes have elapſed,* the blood will alſo begin to ſtagnate *in the veins* of the ſtomach and other abdominal viſcera, or a part of the opium will be conveyed by the lacteals or abſorbents to the heart; and in either caſe (but eſpecially if both theſe occurrences take place about the ſame time, which there is reaſon to believe often happens) a ſmaller quantity of blood will be ſent to the right auricle of the heart, which will immediately take off the plethora, and reduce the frequency and ſtrength of the pulſe, (often conſiderably) below the natural ſtandard; and now a degree of languor will be perceived and be more or leſs conſiderable in proportion to the want of vigour in the circulation, and will be accompanied by drowſineſs, vertigo, pain in the head, nauſea, tremors in the hands, &c.

The duration of theſe ſymptoms will depend upon circumſtances. If only a moderate doſe has been taken, the heart and brain will regain their irritability and mobility in a few hours, and

---

he) *upon ſuch as were addicted to its uſe*, was, that their ſpirits were exhilarated, and from a dozing depreſſed ſtate they became active and alert.

Chardin, in his Travels through Perſia, and the Baron de Tott, in his Memoirs on the Turks and Tartars, ſpeak of its exhilarating effects in ſtill ſtronger language.

The former obſerves, that after the operation of this remedy, *the body grows cold, penſive, and heavy*; *and in this dull and indolent ſituation it remains till the doſe is repeated.* He alſo tells us, that it is found by thoſe who have a diſpoſition for jeſting to increaſe that extremely. In anſwer to which I muſt remark, that this property of *increaſing habitual cheerfulneſs*, does not belong excluſively either to opium or to ſtimulants, the ſame effect being frequently obſerved to reſult from the exhibition of tonics, aſtringents, refrigerants, and even cathartics, *when their operation is ſalutary*; as muſt be well known to every man who has been long in practice. But in the inſtance of opium, I apprehend it muſt often be attributed *to its inebriating properties.*

"The Perſians ſay, it entertains their fancies with pleaſant viſions and a kind of rapture; they very ſoon grow merry, then burſt into a laugh, which continues TILL THEY DIE AWAY IN A SWOON." He had before ſaid, that they accuſtom themſelves to its effects, by beginning with a ſmall quantity, and increaſing it gradually; and he adds, when a Perſian finds himſelf in a diſtreſſed ſituation, he has recourſe to a piece of opium *as big as his thumb*, and immediately after taking this he drinks a glaſs of vinegar; this throws him into a fit of laughter, *which terminates in death.*

* Only forty minutes had elapſed in Dr. Crump's eighth experiment, when he "perceived a pleaſing kind of *languor gradually increaſing*," and the ſcale ſhews, that in forty-five minutes the frequency of the pulſe was *reduced* from 70 to 64. In the 17th experiment, ſixty minutes had elapſed when "a pleaſing languor and drowſineſs came on," but the pulſe had ſunk from 70 to 66 in forty-five minutes. In the 19th experiment, "in thirty-five minutes found a ſlight heavineſs and pleaſing languor coming on, the fulneſs of my pulſe diminiſhing," and in fifty minutes the pulſe was reduced from 72 to 68. Inquiry, p. 35, 65, 66.

and in proportion to the rapidity of this procefs, will they be reftored to the vigorous exercife of their functions, when the languor, drowfinefs, &c. will of courfe difappear; *this procefs however, will proceed much more flowly in the ftomach and inteftines, from the medicine having been applied to them in a more concentrated ftate than to the heart, &c. hence a much longer time will frequently elapfe before they will be capable of performing their functions in a proper manner.*

It too frequently happens, however, (*much more fo than we could have any idea of from the whole tenor of the Inquiry,**) where

* "Thefe experiments, and the authorities above quoted, feem fufficiently to evince, that the primary effect of opium on the pulfe, is to accelerate and render it fuller; and we can only account for the miftake of thofe who maintain a contrary opinion, by fuppofing that they neglected to examine the ftate of the pulfe *fhortly* after the opium had been taken, attending only to the *ultimate* changes it underwent; and this fuppofition will receive further confirmation from confidering, that the only favourer of this opinion, who, as far as I know, has given a particular detail of any experiments in its fupport, has totally omitted any account of the ftate of the pulfe *during the firft half hour*; (Bard Differt. Inaug. de Opio, Edin. edit. an. 1763,) although, as fufficiently appears from thofe above related, its frequency is, during that period, more augmented than during any other, and indeed in a fhorter time than has been generally imagined." Inquiry, p. 36, 37, and 192. It is true that Dr. Crump does once allude to its power of *immediately producing total infenfibility, immobility, and lofs of life, although no fymptom of increafed excitement precedes thefe effects*; (p. 193, 194) but it is rather as an exception to a general rule, than as a circumftance of frequent occurrence, *and he fpeedily and very prudently difmiffes it, as being inconclufive and unimportant.*

There is one circumftance in the experiments of Drs. Crump and Bard, too remarkable to efcape notice.

It appears that in *every experiment* made with opium, *taken internally, of which Dr. Crump has given an account,* the pulfe very foon became more frequent, *and continued fo, (eighty minutes in one of them, viz. the 7th) taking the average of the five experiments forty-eight minutes.* See Exp. 6, 7, 8, 17, and 19.

Dr. Bard made a fimilar experiment *fourteen times*; and it appears from two of them, which are minutely detailed, and from an extract which I fhall make from his work, that he did not *in any inftance delay examining the pulfe longer than half an hour,* (indeed I think it reafonable to conclude from the tenor of his language, that in fome of his experiments he did not delay the examination fo long) *yet he never found its frequency increafed except in one inftance.*

I fhall not attempt to explain this *phyfical prodigy,* farther than by obferving that Dr. Bard's experiments bear evident marks of having been conducted with great circumfpection and accuracy, *and there is no inconfiftency,* that I can perceive, *in his arguments.*

"Hoc experimentum quater in meipfo, pari cum fucceffu, expertus fum. Pulfus fcilicet diminutus, aliaque fymptomata ordine femper redierunt.

"Pari modo, in amicorum tribus, et in fex convalefcentibus nofocomii regii, ejufdem rei periculum feci, et femper iifdem phænomenis redeuntibus. Sed where the dose is large, and the habit unaccuſtomed to opium, or there is a deficiency of vital energy in the frame, that it operates ſo inſtantaneouſly and powerfully as a ſedative upon the ſenſibility, irritability, and mobility, *of the veins as well as the arteries and other veſſels belonging to the ſtomach and inteſtines, and alſo upon the lacteals and ducts which open into the primæ viæ*; *and its influence is ſo much more extenſive than in the former caſe*, as to occaſion a conſiderable quantity of blood, which was returning to the vena cava inferior from the ſtomach, the inteſtines, the ſpleen, the pancreas, the meſentery, *and more eſpecially from the liver*, by the hepatic veins, to be intercepted and detained in the veins belonging to the abdominal viſcera; and one great ſource by which the heart is uſually ſupplied with blood, being in this manner ſuddenly cut off, it will ſend out a ſmaller quantitity at each contraction; and not being able, any more than the aorta and pulmonary arteries, immediately to adapt itſelf to the diminiſhed quantity of blood, the latter will not be propelled with the ſame force as before, which will ſpeedily retard, if not interrupt, the circulation not only in the minute veſſels of the extremities (as we have ſeen exemplified in Dr. Alſton's experiments) but in the whole arterial ſyſtem; the venous circulation will alſo be carried on in a very languid manner; *the proceſſes* of ſecretion and excretion will ceaſe almoſt univerſally; the ſkin will become cold, and loſe its colour; the pulſe and reſpiration will be ſcarcely perceptible; the pupils will be dilated; ſtimulants applied to the organs of ſenſe will produce little or no ſenſible effect. If the vital functions continue long in this ſtate of ſuſpenſion, all attempts to reſtore them will be ineffectual, (the natural functions may be ſuſpended for a much longer time without death enſuing) and the patient will expire without a groan or a ſtruggle; (to adopt the words of Chardin, *he will die away in a ſwoon.* Inq. p. 50) But if they have not been entirely ſuſpended, or have remained only a very ſhort time in that ſtate, and a rational mode of treatment is adopted, the recovery will often be complete.*

But

---

Sed quoniam iis continuo invigilare non poteram, omnia quæ iis contigerunt, ſigillatim enarrare inutile judicavi; in omnibus tamen pulſus ab initio opii actionis tardiores factos deprehendi. Aliter in meipſo tamen ſemel factum fateor; cum enim opii grana duo cum dimidio, devoraſſem; mox per horas duas vel tres, nauſea et vomitu continuo laboravi: qua ſignorum violentia, datique opii copia, nonnihil perterrita, mihi pulſus erant aliquanto citatiores. Quod terrori, et vomitus convulſioni, potius quam alicui opii ſtimulo tribuendum cenſeo; *nam in omnibus, et ante et poſtea inſtitutis, experimentis, pulſus protinus ab initio nunquam non tardati ſunt.*" Bard, de Viribus Opii, p. 17, 18.

* By a rational mode of treatment, I mean ſuch a one as it would be ridiculous to think of, if opium were a ſtimulant; but this is a ſubject too extenſive

But there is a confiderable variety in the fymptoms in different perfons, even in the cafe now fuppofed, as appears from the view taken in my laft paper. In many inftances the ftupor is accompanied by partial or general fpafms, laborious breathing, &c. &c.* all depending upon and eafily traced to, a powerful *and directly fedative caufe*, operating unequally and irregularly upon the irritability and mobility of the vafcular and nervous fyftems.

In treating of the exhilarating properties of opium, I have not enumerated *all* the inftances in which it manifefts fuch a power; nor was it neceffary, becaufe I apprehend *the fame general principles are applicable to every cafe and circumftance whatever, fo as entirely to remove every thing ambiguous and obfcure, relating to its mode of operating.*

Were an impartial obferver to take a view of the experiments which have been brought forward in the courfe of this Effay, he would naturally be defirous to know the *origin* of the ftimulant doctrine, which he muft pronounce to be very inconfiftent and extraordinary. Dr. Crump has given us fome information on this point.

"Galen, extenfive and important to be comprifed in a Note, I fhall therefore decline entering upon it at prefent. A very inftructive cafe is related in the Medical Obfervations and Inquiries, vol. vi. p. 331, and another in Johnftone's Medical Effays, p. 168. Fortunately they fell into the hands of men who were aware of the abfurdity of that doctrine, which would reprefent opium *as operating by a ftimulant power.* Indeed, when I reflect how widely the unfounded and pernicious doctrine juft alluded to, has fpread, what general acceptation it is in, (as appears from almoft every modern medical publication) and that there is too much reafon to apprehend it may become ftill more prevalent, having received the fanction of a late celebrated writer, I am at a lofs in what terms to exprefs my anxiety for the fatal confequences which cannot but enfue.

The Author of a communication "on the ufe of opium in fever," in the 41ft number of the Medical and Phyfical Journal, feems to have entirely miftaken my meaning; (unlefs he meant to fpeak ironically in his note to p. 51, where he thanks me for the pleafure he has received from the perufal of what he is pleafed to term, my *excellent* Differtation on Opium) for if there be any truth in my theory, the opinions held out in his communication are the moft injudicious imaginable; and I confole myfelf with the reflection, that I have not written a fentence which could be fuppofed even to countenance, much lefs to recommend, a practice at all fimilar to that which he inculcates. Thus much I thought it incumbent upon me to fay, to prevent any mifconception which might otherwife go forth.

* "*The mind becomes gradually dull and languid, the body averfe to motion, little affected by cuftomary impreffions, and inclined to fleep. If the dofe has been confiderable, all thefe fymptoms continue to increafe; and tremors, convulfions, vertigo, ftupor, infenfibility, and deprivation of mufcular action, appear varioufly complicated, and in various degrees, proportioned to the excefs in the dofe, and peculiarity of conftitution in the fufferer.*" Inq. p. 44.

"Galen, who seems to have been the first to exhibit opium, though with a sparing and timorous hand, attempted, on those principles by which he was apparently led in his reasonings in other instances, to explain its wonderful, and seemingly deleterious properties."

Medicines, it is known, he divided into *cold* and *hot*; and opium, he asserted, was cold in the fourth or greatest degree. The proofs he assigned in support of this assertion, seem, as far as they can be collected, to have been deduced from its power of inducing sleep, stupor, and other similar affections;* all of which, he asserted, were ever the consequences of the exhibition of cold bodies; and from the nature of those substances which he deemed necessary to be joined with opium in order to correct the supposed intensity of its frigidity; all of which substances he ranked in the class of hot medicines.

Long was the authority of Galen predominant in the schools, both among his cotemporaries, immediate successors, and the first professors of the healing art after the restoration of learning. With his other tenets, this mode of explaining the properties of opium was implicitly acceded to; and one principal consequence resulting therefrom was, that it was always necessary to exhibit with opium some medicines of a hot nature, to correct the intensity of that property by which it was supposed to act. To this circumstance do we owe those famous medicines, the Philonium, Theriaca, and Mithridate; so long from a veneration for antiquity retained, and at length so justly exploded.

As *the Chemists* were the first that openly opposed the authority of Galen and his commentators, so their opinions on many medical questions were nearly opposite. With respect to the nature and mode of action of opium they were diametrically so; for though Galen had placed it amongst the coldest bodies known, the new sect asserted it was of a hot nature, and that *all* its effects were to be attributed to its *heating properties* alone. These positions they supported *by arguments drawn from its ostensible qualities, as its smell, bitter taste, inflammability, &c.* from many of its effects on the human body, as its *exciting heat, thirst, and itching, intoxication*, and other effects always consequent to the exhibition of medicines of a hot nature.

Such were the opposite opinions avowed and supported by these two contending sects, while they continued to divide the medical

* This opinion of Galen, notwithstanding the vague and complex practice to which it gave rise, appears to have been better founded than has been imagined.

medical world. Many, however, were not wanting who steered a middle course, asserting that opium was composed of particles in their nature different, some possessing a hot, and others a cold quality; that the cold were more in number than the hot, and that, in consequence of this composition, the effects which were to be attributed to the hot particles were slight and transient; and those produced by the cold, violent and permanent." Inq. p. 89—92.

I shall now pass on to p. 97, where the following remarks occur.

"By far the greater number of modern authors seem inclined to attribute the effects of opium to its action on the nerves; on which it is generally said to exert an immediate *sedative* power; without its being specified in what particular manner this sedative quality acts; Dr. Cullen, indeed, and others going so far as to say, that sedative powers produce their effects by destroying the *mobility* of the nervous fluid. Yet, as among others, many effects are observable from the action of opium, which are by all attributed to bodies termed *stimulant*; many of these authors are inclined to attribute to it a stimulant quality also, but of so slight and transient a nature, as to be soon overcome by its opposite, or the sedative power."

"The stimulant powers of opium are, indeed, in numerous instances, so remarkable, that we find many writers comparing its mode of action and effects to those of substances *universally reckoned powerful stimulants, as wine, alcohol, and the whole class of cordials*;* although its remarkable pre-eminence in allaying pain and irritation, and producing sleep, insensibility, &c. has induced them, when attempting to explain its action, to ascribe to it a direct and powerful sedative quality. *But the only writers or teachers, who, as far as I know, have expressly denied any direct sedative power in opium, and ascribed all its effects to its stimulant quality alone, are the late Dr. Brown, in his Elementa Medicinæ, Lectures, and other productions, and his Pupils.* Opium according to this Author, like every other medicine,

---

* This vague method of reasoning occurs very frequently in this and the succeeding Chapters, See p. 164, 5; 170, 171; 177; 195—202. It is exceedingly objectionable, as I have already endeavoured to shew (M. & P. J. v. vii. p. 127.) *It is not true that wine and alcohol are universally reckoned powerful stimulants*: (See Cull. Mat. Med. v. ii. p. 315—317,) and what is meant by the whole class of cordials?

Neither does it follow, because opium acts in the same manner as wine and alcohol, that it is therefore a stimulant. So loose a method of arguing would be rejected in discussing almost any other subject; and is not accuracy as much required (to say the least) in a physical discussion, as in any other?

medicine, acts primarily on the living principle, or, as he terms it, excitability of the syftem; *is poffeffed of a highly ftimulant quality, and allays pain, induces fleep, intoxication, and other fimilar affections,* BY THE EXCESS OF THIS POWER ALONE. ITS REMARKABLE PRE-EMINENCE, HOWEVER, AS AN ANODYNE, OVER OTHER STIMULANTS, HE HAS NOT, AS FAR AS I CAN COLLECT, ATTEMPTED TO EXPLAIN." Inq. p. 97—99.

This honour appears to have been referved for Dr. Crump; and it will be very eafy to fhew, when I come to the arguments he brings forward in fupport of this prepofterous notion, that he poffeffed a fufficient portion of zeal to qualify him for fo extravagant a project.

Such was his ardour in the caufe, that he has travelled (in idea) *nearly to the north pole* in fearch of arguments; (See p. 163—4) and *elucidates* his theory by what he calls a few *appofite* examples, deduced from *analogous* facts with refpect to fenfation.

Thefe *appofite* examples confift in fhewing the effects of expofing THE EYE TO VERY BRIGHT SUN SHINE, and fuddenly removing the perfon who is the fubject of the experiment *into a dark room:* 2dly, expofing the whole body, or any particular part of it, *to a high degree of heat, and fuddenly reducing it* 20 *degrees.* (P. 189—90.)

Another of thefe *appofite* examples is to be met with, p. 164. "If the hand immerfed in water of confiderable warmth, be taken out and plunged in a veffel containing a quantity of the fame fluid of a fomewhat lower temperature, inftead of the fenfe of heat which would have arifen, were it not for its previous application in a more confiderable degree, that of cold will be the confequence."

Such bold flights may fhew the ingenuity of the author, and may deceive by their fpecioufnefs, but will not bear the teft of fober inquiry.

"Another opinion which remains to be mentioned, is that lately advanced by the Abbe Fontana. From a number of experiments made with opium on different animals, he pofitively afferts that it has no action whatever on the nervous fyftem, or vital folids; and that all its effects are to be attributed to changes induced by it on the blood. The particular nature, however, of thefe changes, he appears neither to attempt to fpecify, nor to anfwer the many objections urged by preceding writers againft explanations of a fimilar kind."

"Such appear to have been the principal theories which have prevailed on the fubject we are engaged in; and, though each in turn has had its numerous advocates, we find many phyficians cians ſo far diſſatisfied with the explanations reſulting from them, as ever to be ready in acknowledging their ignorance with reſpect to its real operation, and willing to attribute the effects it produced to ſome peculiar and undiſcovered properties; and, notwithſtanding an explanation of its action has been attempted by a conſiderable proportion of the many medical writers who have flouriſhed ſince the very firſt ages of the ſcience, as appears from the preceding part of this chapter; yet it will be ſufficiently evident, from a little conſideration, that the ſeeming variety of opinions on the ſubject, however aparently different, may be reduced, with propriety, to three claſſes only. The firſt, containing the opinions of thoſe who aſcribe its effects to changes induced by it in the blood. The ſecond, of thoſe who deduce them from its action on the living principle as a ſedative, or ſedative and ſtimulant conjoined. And the third, comprehending the ſentiments of ſuch as attribute to it the properties of a ſtimulant alone."

"For in what material circumſtance do the diſputes, with reſpect to its *hot* and *ſtimulant*, its *cold* and *ſedative* qualities, materially differ? Are not the ſentiments of many moderns, who deem it poſſeſſed of both a ſtimulant and ſedative power, ſimilar to thoſe of more ancient phyſicians, who aſſigned to it hot and cold qualities in different proportions? And will not a little attention ſufficiently evince, that the mechanical conjectures of many, as to the particular manner in which it was ſuppoſed to obſtruct the nervous canals and motions of the animal ſpirits, do not vary eſſentially from the ſentiments of thoſe moderns, by whom its ſedative effects are aſcribed to a diminiſhed mobility of the nervous fluid?" Inq. p. 99—102.

The above extracts ſhew the ſituation of the controverſy at the time Dr. Crump entered upon it.

Chapter V. is occupied in refuting the opinions of thoſe who aſcribe the operations of opium to changes induced by it in the fluids, and in conſidering the arguments adduced to prove that the nervous ſyſtem is inſenſible to the operation of opium, and the blood alone liable to its action: opinions ſo ill founded as ſcarcely to have merited a ſerious refutation.

In the VIth Chapter, Dr. Crump enters into an examination of the different theories which have been advanced to explain the phenomena ariſing from the exerciſe of the nervous functions.

I have very few obſervations to make upon this part of the Inquiry. For a more copious account, ſee p. 144—153.

"From the firſt commencement of any controverſy upon this ſubject, the prevailing opinion ſeems to have been, that the immediate agent in all the nervous or animal functions was a peculiar a peculiar fluid fecreted into, and freely flowing through the cavities, or inherent in the fubftance, of the nervous fibres. This precedence it ftill retains, and, as the only explanation at prefent offered, feems alone to merit a particular difcuffion. To me I muft confefs, it appears no better founded than any other advanced upon the fubject, and I fhall briefly ftate the reafons which have induced me to form this opinion." P. 143.

Here I entirely coincide with Dr. Crump. Indeed, as no appearance of a cavity is difcoverable in the nerves, even with the aid of a microfcope, one would imagine there could fcarcely be two opinions on the fubject.

Befides, fuppofing the theory of a nervous fluid to be juft, it would ftill be imperfect, as appears from what follows.

"This ftructure is furely incompatible with the opinion of fenfation being occafioned by the nervous fluid moving towards the brain, and mufcular actions depending on its motions from it; both functions are frequently exercifed together, and we can hardly fuppofe that a fluid moves at one and the fame time through a long, extremely minute, and varioufly inflected cavity, in oppofite directions, without irregularity or confufion. If the nerve which fupplies any particular limb or mufcle, be divided and preffed between the fingers from its infertion in the mufcle towards its divided extremity, the fibres it fupplies, as I have myfelf often experienced, will be thrown into convulfive actions; but if the action of a mufcle be the confequence of the nervous fluid's being urged in unufual quantity into its fibres, we can hardly fuppofe that fuch effects would follow from an experiment, whofe only tendency, did fuch a fluid exift, would be to move it an in oppofite direction. Similar convulfive actions will enfue, if the nerve of any mufcular part be irritated by a fharp and penetrating inftrument." Inq. p. 149—151.

But I am by no means fatisfied with the hafty manner in which Dr. Crump has difmiffed the theory of vibration. He merely tells us, that the hypothefis has been refuted by very fufficient and convincing arguments, and that it feems at prefent to be *univerfally* deferted.

They muft have more faith, however, in Dr. Crump's decifions than falls to my fhare, who can reft fatisfied with the information here afforded.

*Whatever may be the nature or effence of the nervous power*, *every experiment hitherto made on the inferior animals*, at leaft every one I have met with) *fhews, that it is fo much diminifhed by a moderate dofe of opium, that the nerves are with difficulty made to obey a ftimulus*; *and the effect is the fame, as far as its influence extends, to whatever part of the body the application is made*,

*made*, (except it be to the nerves themſelves:) *but is moſt general and apparent when injected into the ſtomach of the animal: and that it is ſo immediately and completely deſtroyed by a large doſe, that the nerves, as well as the muſcular fibres connected with them, become totally inſenſible to every kind of ſtimulus*; NOR IS THIS EFFECT PRECEDED, IN EITHER CASE, BY ANY SYMPTOM OF INCREASED EXCITEMENT, *which it ought to be, were opium a ſtimulant.*

I ſhall not ſtop, now, to inquire into the merits or demerits of Dr. Crump's theoretical reaſonings, which occur next in order; but ſhall paſs on to his concluſions. P. 191—193.

"To render my ſentiments upon this ſubject as perſpicuous as poſſible, I ſhall next endeavour to bring them under one clear point of view, by reducing the whole of the concluſions to be deduced from the preceding facts and arguments, to a few general heads; and the following appear to me to comprehend them all.

1. Opium is a medicine endowed with a ſtimulant property, conſiderable in degree, readily diffuſible over the whole ſyſtem, and eaſily and ſuddenly exhauſted.

2. In conſequence of the immediate operation of this ſtimulant power, the pulſations of the heart and arteries are rendered quicker and ſtronger; the heat of the body is augmented, its perſpiration promoted, ſweating ſometimes excited, the mind exhilarated, the delirium of intoxication (if the medicine be taken in ſufficient quantity) induced, ſuch morbid ſymptoms as ariſe from general debility removed, and the excitement, in ſhort, of the whole ſyſtem increaſed.

3. But this ſtimulant power being ſuddenly exhauſted, the ſyſtem at large is left inſenſible, in a great degree, to the operation of every exciting cauſe; and as thoſe continuing to act are generally inferior in point of exciting force to that which the ſyſtem was ſo ſuddenly deprived of, their effects are rendered leſs conſiderable than before its operation: hence the pulſations of the heart and arteries become ſlower; pain and inordinate action, as ariſing either from a morbidly acute degree of ſenſibility, or the preſence of irritating powers, are alleviated or totally removed; watchfulneſs, as produced by ſimilar cauſes, prevented or ſuppreſſed; the mind is rendered dull and languid; and if the doſe has been conſiderable, INDIRECT DEBILITY ſucceeds; ſtupor, tremors, and other ſimilar ſymptoms enſue; the pulſe intermits, AND DEATH perhaps concludes the ſcene."

Theſe concluſions are immediately ſucceeded by the following remarks, *which plainly ſhew, that Dr. Crump's mind was ſo completely*

*pletely biaſſed by prejudice, as to have diſqualified him for the inveſtigation of phyſical truths**.

"With reſpect to the objections which may be made to this explanation of the properties of opium, the only one which can, as appears to me, be urged againſt it, *and which has not been anſwered, is that founded on* AN UNCONTROVERTIBLE FACT, *viz. that opium applied to different parts of living animals, is* IMMEDIATELY *productive of* TOTAL INSENSIBILITY, IMMOBILITY, AND LOSS OF LIFE, ALTHOUGH NO SYMPTOM OF INCREASED EXCITEMENT PRECEDES THESE EFFECTS†. *It has thence been concluded, that the medicine operates in every inſtance by a directly ſedative quality; and as it is the principal argument relied on in attempting to prove this opinion, and may be adduced againſt thoſe in this chapter adopted, I have conſidered it with particular attention,* BUT FIND IT NOT POSSESSED OF ANY FORCE, NOR IN THE LEAST DEGREE CONCLUSIVE; AND FOR ONE *ſimple*, BUT VERY SUFFICIENT REASON, WHICH IS, THAT MANY OTHER ARTICLES, *univerſally* ACKNOWLEDGED TO BE ENDOWED *ſolely* WITH A STIMULANT PROPERTY, ARE PRODUCTIVE, IN SIMILAR CIRCUMSTANCES, OF THOSE EFFECTS WHICH, WHEN INDUCED BY OPIUM, ARE RECKONED THE CONSEQUENCES OF A DIRECTLY SEDATIVE QUALITY ALONE. *A few of the experiments I made with a view of determining this point, it may not be amiſs to ſtate: They were indeed among the firſt I inſtituted on the ſubject, at a time when I was engaged in conſidering the validity of the uſual arguments advanced in ſupport of the generally received opinion with reſpect to the ſedative qualities of the medicine.* THE STIMULANTS I EMPLOYED WERE, RECTIFIED SPIRIT OF WINE, THE VOLATILE ALKALI, AND ELECTRICITY, THREE OF THE MOST

---

* "*Of the numbers employed in the purſuit or practice of Medicine,* (ſays Dr. Crump, Introduction, p. 3, 4.) *a great part muſt, of courſe, be deficient in the ſtrength and acuteneſs of genius neceſſary to decide with propriety between a diverſity of opinions:* SUCH HAVE ALWAYS BEEN INVINCIBLY PREPOSSESSED IN FAVOUR OF IDEAS FIRST IMBIBED, &c."

† *Here Dr. Crump acknowledges that his theory is at variance with an uncontrovertible fact; one too of frequent occurrence.* (See his 12th, 30th, 32d, 40th, and 42d experiments.—Supplement to Fontana's Treatiſe on Poiſon.—Willſon on Opium.—Alexander, Differt. inaug. de partibus corporis animalis quæ viribus opii parent.) *and uncommon importance,* AND WHICH IS DECISIVE IN PROVING THE STIMULANT THEORY TO BE FALLACIOUS AND UNTENABLE; *but rather than abandon it, which it is evident he muſt have done, had the diſcovery of truth been his chief object, he had recourſe to the only remaining expedient—evaſion.*

POWERFUL *we* ARE ACQUAINTED WITH*; *and my intentions at the time were to determine whether, when applied to animals in a similar manner with opium,* THEY WERE NOT EQUALLY EFFICACIOUS IN DESTROYING ALL ACTIVITY AND SENSIBILITY."—Inq. p. 193—5.

*Here Dr. Crump evidently shifts his ground, and the question is no longer whether opium acts as a stimulant or a sedative; but whether* OTHER ARTICLES, *which he enumerates, and which he says,* (with what truth will appear by referring to Cullen's Mat. Med. vol. ii. p. 315. 317) *are universally acknowledged to be endowed* SOLELY *with a stimulant property; are not equally as efficacious as opium,* IN DESTROYING ALL ACTIVITY AND SENSIBILITY.

This had been proved, by experiments conducted upon a much more elaborate plan than those of Dr. C. *long before his publication appeared* †; *but so far is this circumstance from proving opium to be a stimulant, that it establishes, beyond a controversy,* A POSITION EXACTLY THE REVERSE; *viz.* THAT NOT ONLY OPIUM, BUT ALSO SPIRIT OF WINE, VOLATILE ALKALI, AND STRONG SHOCKS OF ELECTRICITY, ACT DIRECTLY AS SEDATIVES ‡.

The experiments alluded to in the passage just quoted, are as follow:

Expt. 32. "Having separated by inflation, the skin and muscles of one of the posterior extremities of a frog, I poured between them some of a strong watery solution of opium. THE LIMB WAS DEPRIVED OF ALL SENSIBILITY AND POWER OF MOTION *in ten minutes.*"

---

* This is a declaration which I sincerely hope, none but a disciple of the Brunonian school could have made. *They certainly act very powerfully; but not as stimulants, as will be seen by and by.*

† Essays and Observations Phys. et Lit. vol. iii. p. 340—358.—Fontana on Poisons, Supplement, vol. ii.—Alexander, Dissert. Inaug. de Opio.

‡ *To the same class undoubtedly belong* THE VENOM OF THE VIPER, THE TICUNAS, AND THE CHERRY LAUREL. From an attentive examination of the experiments made by the illustrious Fontana with these poisons, and comparing them with the experiments made by himself (see his Supplement) and others, *with opium, spirit of wine, &c.* I am fully persuaded (and whoever will investigate the matter *with a mind free from prejudice,* will, I think, be of the same opinion) THAT THEIR MODUS OPERANDI IS DIRECTLY AND POWERFULLY SEDATIVE; but I am under the necessity of deferring the farther consideration of this important subject, together with the mode of treatment, which this idea of their manner of operating suggests, to another Number of the Medical and Physical Journal; when I hope to shew, in a tolerably satisfactory manner, that the numerous experiments made by this truly great philosopher, may possibly at length, *in some degree,* answer the purpose intended by their benevolent author.

Expt.

Expt. 33. "Between the ſkin and muſcles of the poſterior extremity of another, I poured a quantity of *ſpirit of hartſhorn, which was productive of the ſame effects as opium in the former experiment in about* ONE *minute.*"

Expt. 34. "*Employing* RECTIFIED SPIRIT OF WINE in the ſame manner as the other articles in the two preceding experiments, *I found it ſucceeded*, by ſimilar conſequences, in* THREE *minutes.*

Expt. 35. "A SMART SHOCK OF ELECTRICITY having been paſſed through the hind leg of another frog, IT INSTANTLY RENDERED IT COMPLETELY PARALYTIC.

Expt. 36. "Having taken out the heart of a frog, and immerſed it in ſome of the ſame ſolution of opium employed in Experiment 32, I FOUND IT DEPRIVE IT OF ALL POWER OF CONTRACTION *in* TEN *minutes.*

Expt. 37. "The heart of another, immerſed in *ſpirit of hartſhorn*, WAS REDUCED TO A SIMILAR STATE *in* TWO *minutes.*

Expt. 38. "RECTIFIED SPIRIT OF WINE DEPRIVED A THIRD OF ALL POWER OF MOTION IN THREE MINUTES.

Expt. 39. "Having perforated a piece of common pane glaſs with a ſmall hole, I laid over it the heart of a frog, *and through it ſent* A SMART SHOCK *of Electricity*, WHICH INSTANTLY DEPRIVED IT OF ITS CONTRACTILE POWERS. I took the precaution of uſing the perforated glaſs, as I found the electric fluid was otherwiſe liable to glide over the ſurface of the heart, probably from its moiſture, without affecting it in any conſiderable degree.

I muſt obſerve, however, that the ſhocks I employed were not ſo vehement as to injure the organic ſtructure of the parts on which theſe experiments were made."

"The preceding Experiments, *which I have frequently repeated*, but with ſo little variation that a particular account ſeems not requiſite, *ſufficiently ſhew, that three of the* MOST POWERFUL STIMULI *we* ARE ACQUAINTED WITH ARE CAPABLE OF PRODUCING THOSE EFFECTS ON THE PARTS TO WHICH THEY ARE IMMEDIATELY APPLIED, WHICH, WHEN INDUCED BY OPIUM, WERE DEEMED THE CONSEQUENCES OF ITS SEDATIVE POWER; NAY, THAT THEY ARE PRODUCTIVE OF THESE EFFECTS EVEN MORE SPEEDILY THAN OPIUM ITSELF; AND IT CONSEQUENTLY APPEARS, THAT ANY ARGUMENTS DEDUCED FROM THESE OR SIMILAR FACTS, WITH AN INTENTION OF PROVING THE EXISTENCE OF THAT SEDATIVE QUALITY, IS INCONCLU-

* *In depriving the limb of all ſenſibility and power of motion.*

SIVE*. With a view of comparing *the effects of theſe ſtimuli upon the heart*, the following experiments were made."

Expt. 40. " Having extended the limbs, and tied down a large frog upon its back, I opened the thorax juſt ſo as to bring the heart into view; its pulſations were 62 in a minute. I then injected into the anus, by means of a ſmall ſyringe, *ſome rectified ſpirit of wine*; but the quantity of which could not be exactly determined, as ſome of what had been injected was again diſcharged. Leaving the animal in this ſituation, I attended to the changes induced in the heart's motions; and reckoning its pulſes at the periods mentioned in the firſt line of the following table, found their number to be as is ſet down in the ſecond:

| In | 2 | 5 | 10 | 15 | 20 | 25 | 30 | 35 | 40 | 45 | 50 | 55 | 60 minutes |
|---|---|---|---|---|---|---|---|---|---|---|---|---|---|
| The P. was | 62 | 62 | 62 | 52 | 46 | 44 | 42 | 40 | 38 | 36 | 34 | 32 | 32 |

Sixty minutes elapſed, the animal was, in external appearance, dead, although the heart ſtill continued to beat, and was thrown out.

Expt. 41. " Having tied down a frog, as in the former Experiment, brought its heart into view, whoſe pulſe was 50 in a minute, and injected *ſome ſpirits of hartſhorn*, I found it affected in the following manner:

| In | 5 | 10 | 15 | 20 | 25 | 30 minutes |
|---|---|---|---|---|---|---|
| The Pulſe was | 50 | 30 | 22 | 6 | 5 | 3 |

Expt. 42. " Into a third, whoſe pulſe was 58 in a minute, I injected ſome of *a watery ſolution of opium*, and found it affect the pulſe in the following order:

| In | 2 | 5 | 10 | 15 | 20 | 25 | 35 | 40 | 45 | 50 | 55 | 60 minutes |
|---|---|---|---|---|---|---|---|---|---|---|---|---|
| The P. was | 56 | 50 | 46 | 38 | 37 | 35 | 34 | 32 | 32 | 32 | 32 | 32 |

The animal was ſtill poſſeſſed of ſome power of motion and ſenſibility, but in ſo trifling a degree that he was killed and thrown out.

* There never was, perhaps, a ſtronger proof of inconſiſtency and abſurdity than this inference exhibits. Indeed, had Dr. C.'s mental faculties been as much under the influence *of theſe powerful ſtimuli, as he calls them*, as the bodies and limbs of the frogs uſed in the laſt eight, and in the three following experiments, *they could not, apparently, have been more completely deprived of their ſenſibility and energy.*

Expt.

Expt. 43. " Having tied down a frog and brought the heart into view, whose pulsations were 70 in a minute, I passed *a smart shock of Electricity* through the whole body from its head to it lower extremities. *The pulsations of the heart* INSTANTLY *ceased, and it contracted but once when irritated with a needle.* Leaving the animal in that situation, I observed, that in about five minutes, *the heart began again to beat*, FIRST SLOWLY, BUT WITH INCREASING CELERITY, *till its pulsations rose to* 35 *in a minute. As they did not increase above this number, I gave another shock similar to the first, which was* IMMEDIATELY *productive of like effects; and after eight minutes more had elapsed, I could not perceive the smallest sign of returning pulsation. I then began to draw* THE GENTLEST SPARKS *from the heart, and was surprised to perceive it again begin to move; and its contractions, by continuing this application*, INCREASED GRADUALLY, TILL THEY AMOUNTED TO 31 IN A MINUTE; *when giving the animal a third shock, it entirely deprived it of life, for no farther application of any kind could rouse its heart into the smallest motion.*" Inq. p. 195—201.

All writers of credit, however they may differ in other respects, *agree in defining stimulants to be such articles as* INCREASE *the animal energy**; and Dr. Crump evidently subscribes to this definition †. *The most powerful stimulants might therefore be expected to increase the motions and powers of motion*, either generally or partially, according to the manner of applying them, *pretty considerably. We may therefore conclude, that if opium, spirit of wine, spirit of hartshorn*, AND STRONG SHOCKS OF ELECTRICITY, *are stimulants of the most powerful kind, as Dr. Crump asserts, he must have applied them improperly; for it appears from five of his Experiments* (the 12th, 32d, 33d, 34th, and 35th,) *that the part to which they were applied*, WAS ALMOST IMMEDIATELY DEPRIVED OF ITS SENSIBILITY AND MOBILITY, AND WAS THE ONLY PART OF THE BODY THE ANIMAL WAS UNABLE TO MOVE. *They had a similar effect upon the hearts of frogs separated from the body in Experiment* 30, 36, 37, 38, and 39; *and where they were applied so as to affect the system generally*, as in Experiments 40, 41, 42, and 43: THE FIRST ALTERATION WAS A DIMINUTION IN THE FREQUENCY OF THE PULSE, *which became slower and slower*, TILL THE ANIMALS WERE REDUCED TO A STATE

---

* Culs. Mat Med. vol. i. p. 187; vol. ii. p. 131, 132.—Brown's Elements, vol. i. p. 6.—Darwin's Zoonomia, vol. i. p. 13. 30. 73 and 74.

† See Inq. p. 160, 161. 169, 170. 184, 185.

OF INSENSIBILITY AND IMMOBILITY; *to which ſtate they were brought in a fourth part of the time they would have been, had their heads been cut off, and the ſpinal marrow deſtroyed*; and I do not ſuppoſe any one will aſſert, that decollation acts as a ſtimulant; and yet it acts in the ſame manner as the articles above enumerated, in immediately diminiſhing, and ultimately deſtroying, the ſenſibility, irritability, and mobility of the body.*

That theſe reflections did not occur to Dr. Crump, is eaſily accounted for, from his being ſo ſtrongly attached to preconceived ideas.

He is peculiarly unfortunate in his concluding remarks, p. 201—206.

"*From the firſt Experiments related in this chapter, it appears, that ſpirit of wine, and the volatile alkali,* DESTROY *even more ſpeedily than opium,* THE MOBILITY AND SENSIBILITY OF THE PARTS TO WHICH THEY ARE IMMEDIATELY APPLIED. The ſucceeding Experiments ſhew, that although opium, from its more ready diffuſibility, *affects* the pulſe *ſooner* than either of them†; *yet that they at length are productive of ſimilar effects, if applied in conſiderable quantity; and that the moſt powerful ſtimuli* ‡, *if applied to the human frame in immoderate doſes, are, as well as opium, capable of inducing ſtupor, inſenſibility, ſlowneſs of pulſe, and other ſimilar affections, is evident, from many caſes in which an immoderate quantity of ſpirituous liquors have been taken at once, and which have frequently terminated in death, preceded by thoſe ſymptoms which an over doſe of opium is generally productive of.*"

---

* Eſſ. and Obſ. P. and L. v. ii. p. 308. 311, 312, and vol. iii. p. 297. *Pugnat ſententia ſecum,* (the phraſe made uſe of by Dr. Crump, p. 125, in reviewing Dr. Corrie's Eſſay on the Vitality of the Blood, *may be applied with propriety to many parts of the Inquiry: On the preſent occaſion it is peculiarly applicable.*

† Compare Expts. 40, 41, 42.

‡ *Thoſe articles which Dr. Crump, in defiance of reaſon and probability, ſo often calls by this name, do not act as ſtimulants;* BUT DIRECTLY AS SEDATIVES: See his 12th. 30th, 31ſt, 32d, 33d, 34th, 35th, 36th, 37th, 38th, 39th, 40th, 41ſt, 42d, and 43d *experiments. Dr. Monro has proved, by experiments upon frogs,* THAT BRANDY, AND ALSO CAMPHIRE, ACT DIRECTLY AS SEDATIVES.—Eſſays and Obſ. Phyſ. and Lit. vol. iii. p. 340—358; *and Dr. Cullen has ſhewn, by the moſt uncontrovertible arguments, as well as by indiſputable facts, that they operate in the ſame manner* UPON THE HUMAN SUBJECT—Mat. Med. v. ii. Art. Sedantia. *It appears alſo from Fontana's experiments upon guinea pigs, turtles, frogs, and leeches,* (which were publiſhed before Dr. Crump's) *that ſpirit of wine acts directly and powerfully as a ſedative.*—See his Supplement; *and the ſame fact is alſo proved by Dr. Alexander's experiments upon frogs and dogs.* Diſſert. Inaug. de Opio.

*The only other fact I shall consider, as demonstrating that the same substance, which in a moderate quantity is capable of exciting the living principle of animals, will, if exhibited in an over proportion, totally destroy their excitability, is the well known property which Electricity possesses of inducing paralytic affections if applied in extremely violent shocks, and of removing the very same complaints if employed in more moderate proportion.* This is particularly illustrated by a fact noticed by Fontana, and further confirmed by the experiment above related.—"Electricity," says he, "induces death by destroying the irritability of the heart and muscular fibres; and yet this same Electricity is, notwithstanding, one of the strongest stimulants to the muscular fibres that is known. It restores life by exciting irritability, in the very animals in which it had, an instant before, destroyed it. Amongst all the stimulants that can be employed to call the animals back to life, that the electrical shock has thrown into a state of insensibility, a proper application of gentle sparks appears to me the most efficacious remedy."—Skinner's Translation, vol. i. p. 96.

It is evident both from the observations of Fontana, and from Dr. Crump's 43d experiment, *that violent shocks of Electricity directly diminish and destroy the animal energy, and that gentle sparks revive and increase it, or, in other words, that electricity may be so applied as to be made to act either as a sedative or a stimulant; but it remains to be proved that any of those medicines which Dr. Crump stiles powerful stimuli, can be so applied as to be made to produce these contrary effects.*

*It appears, indeed, from a great number as well as from a great variety of experiments, that in whatever proportion opium is applied to the bodies of animals,* THE EFFECTS ARE THE SAME IN KIND, DIFFERING ONLY IN DEGREE. *A few examples may suffice.*

Dr. Monro first poured *forty* drops of a solution of opium under the skin of the left thigh and leg, *in three frogs.* He did not attend to the effects till half an hour after, *when he found that whole member paralytic, its toes and skin having lost their sensibility, and the muscles their motion.* The animal seemed much stupified, and could scarcely move its body by the help of the other hind extremity; *and the blood had ceased from motion in the small vessels of both feet,* though, on examining the heart, he found it still gave 22 regular but feeble strokes in the minute. In ten minutes more, the animal became convulsed and died.

( TWO TRIALS. )

Dr. M. then tried the same experiment with *twenty* drops only of the solution; and observed, that in about *a quarter of an hour that extremity was much weakened and less sensible, and* in

*in five minutes more was not only motionless and insensible, but the animal seemed to be much stupified and lay still, unless when it was hurt; and after nearly the same time as those above, became convulsed and expired.*

( TWO TRIALS. )

After that, he tried the same experiment with *ten* drops only. *After twenty minutes that leg seemed to be weaker, and in ten minutes more its muscles lost their power, and the toes had little sensibility; and now the animal seemed to be a good deal stupified, and the heart of one of them examined gave only* 25 *strokes in a minute. An hour and a half after the beginning of the experiment, the toes seemed to have quite lost their sensibility, and the muscles their motion; but the other parts were not convulsed, and the animal jumped by the help of the other hind extremity. By degrees it recovered both sense and motion.*

( TWO TRIALS. )

In the last place, Dr. M. poured *ten* drops under the skin of the thigh only in one frog, and the like quantity under the skin of the leg only in another, *without finding that either of these members lost their sense or motion, or that the rest of the body was observably affected.* Eff. et Obf. P. et L. Vol. iii. p. 309—312. See also Med. and Phys. Journal, vol. vii. p. 350.

I shall transcribe Dr. Monro's first experiment with ARDENT SPIRITS, not because it is more important than the rest, but as being more adapted to the present purpose.

( FOUR TRIALS. )

" *About seventy drops of a mixture of equal parts of* WHITE WINE AND FRENCH BRANDY *were applied on lint to the hind extremities of a frog. In seven minutes the animal was observably affected,* AND ITS HEART BEAT ONLY FIFTY TIMES IN A MINUTE. *In a quarter of an hour the heart beat only forty-five times in a minute; yet the rapidity of the blood in the hind feet was not very observably lessened. After twenty minutes the heart beat only forty times in a minute, and the creature was much stupified. After half an hour the heart beat only thirty-five times in a minute, and evidently with less force than natural; now the circulation was considerably less rapid in the vessels of the hind feet, and the creature was scarcely able to withdraw its legs when the toes were pinched. After three quarters of an hour the heart contracted but thirty times in a minute, and the motion of the blood in the vessels of the hind feet was extremely slow, and ceased altogether after fifty-five minutes; at which time the creature was unable to withdraw its legs from the most severe injury; or when the eye-ball was fretted with a probe, to shut its eyes, which were wide open; and its respiration had entirely ceased.*

I now

I now removed the mixture and wafhed the legs in water; *notwithftanding which it remained infenfible for about an hour and a half longer, although the heart continued the whole time to vibrate, feebly indeed, about thirty times in a minute. But after this it began to bend the joints of the hind legs fpontaneoufly; and in forty minutes more was able to fcramble over the edge of the plate on which it was laid; yet, till half an hour after, when the heart contracted forty times in a minute,* THE PROGRESSIVE MOTION OF THE BLOOD IN THE FEET WAS NOT RENEWED. *Thus the animal gradually recovered its fenfibility and motion, and the blood its free circulation; after three hours more, or about the end of the feventh hour from the experiment, it could jump with almoft its wonted vigour, and the motion of the blood in the feet appeared free and rapid.*

*When the mixture was applied to one leg only, that leg feemed to be fomewhat, but not very confiderably, more benumbed than the other; yet the circulation did not ceafe fooner in the one than in the other leg.*" Eff. p. 340—342. Compare Dr. Alexander's 20th, 21ft, 22d, 23d, 42d, and 43d experiments.—See alfo Wilfon on Opium, p. 92—3.

Dr. Crump permitted an excellent opportunity to efcape, of afcertaining how far he was correct in fuppofing thofe fubftances which were employed in his 40th, 41ft, and 42d experiments, viz. fpirit of wine, fpirit of hartshorn, and opium, to be endued with a power fimilar to that poffeffed by the electric fluid, of deftroying the action of the heart when exhibited in an over proportion, and afterwards of exciting and increafing its action when applied in a moderate quantity; I mean, by injecting *after the heart had ceafed to act*, a fmall quantity more fp. of wine, fp. of hartfhorn, or of the folution of opium, in the gentleft manner, either per anum as before, or into the auricle or ventricle of the heart. But, indeed, the experiment might ftill be made. If it fucceeds *in reftoring the action of the heart*, which I believe few will expect, it will prove that thefe fubftances act either as fedatives or ftimulants, according to circumftances, *which Dr. Crump does not allow to be the cafe with regard to opium*; (p. 167—169, &c. &c.) if it does *not* fucceed, it will add another highly culpable inftance of inaccuracy to thofe brought againft Dr. C.

The fixth Chapter of the Inquiry concludes as follows:

" Having endeavoured to obviate fuch objections as may be urged againft the explanation fupported in the preceding pages, it may be deemed fuperfluous to attempt a further vindication of fuch an inquiry. But it has been by fome remarked, that any inveftigation of the mode of action of medicines ufually termed fedative is unneceffary; for that as they are only exhibited bited with a view to their ultimate effect, it matters not in what manner these effects are brought about, or whether it be determined that they are the consequences of a preceding stimulant exertion or not."

Such I shall answer in the words of the immortal Bacon, by whom mankind, first extricated from the labyrinths of imagination, were conducted to the direct and only true paths to knowledge, and whose injunctions should be strictly followed in every scientific research. Hoc ipsum," says he, "*prius* et *posterius* in omni actione naturali notare debet."* A caution to be especially kept in view where the primary action of a medicine is so obvious and important as that of opium; and the neglect of which is so strikingly censured, and its observance in every instance so strongly recommended by the same author in the following words: "Mira est hominum circa hanc rem indiligentia; contemplantur siquidem naturam tantummodo desultoriè, et per periodos, et postquam corpora fuerint absoluta, et completa, et non in operatione sua. Quod si artificis alicujus ingenia et industriam explorare et contemplari quis cuperet, is non tantum materias rudes artis, atque deinde opera perfecta conspicere desiderat, sed potius præsens esse, cum artifex operatur, et opus suum promovet: Atque simile quiddam circa naturam faciendum est."†

It will be unnecessary to enter into any further examination of Dr. Crump's *hypothetical reasonings, since it must plainly appear from the copious extracts made from his work, that these, as well as his conclusions, are inconsistent with his facts; of course, they must fall to the ground.* It will be proper, however, to point out a few instances of misrepresentation, which occur p. 172, 173.

"And first (says Dr. C.) let me place the venerable Sydenham, in general sagacious in his inquiries, and ever actuated by the spirit of fidelity in relating their results: Engaged in extensive practice, this medicine was frequently exhibited by him, and in so great a variety of instances, his attentive mind *could scarcely fail being struck with the stimulant powers it so obviously possesses*; and we accordingly find, that he not only frequently prescribed it with an intention of supporting the powers of nature when languishing or oppressed, but considered it as the most supreme cordial ever discovered: "Et præstantissimum sit remedium,

---

* Novum organum, lib. 2. aph. 46. circa finem. It would have been prudent in Dr. C. to have refrained from this display of his motto, as it does not appear from his work that he paid much regard to the instruction it contains.

† Ib. Aph. 41.

remedium, *cardiacum* unicum pene dixerim,"* are the expreffive words he employs in conveying this fentiment to his readers. † That the celebrated Cullen perceived fimilar effects, and prefcribed it with fimilar intentions, will be evident from a flight perufal of his practical works. ‡ In Haller's Commentaries on the Inftitutes of Boerhaave, we meet with a paffage *which clearly proves that he alfo was ftruck with its ftimulant properties, as he therein compares its action to one of the moft powerful ftimuli we are acquainted with.*" "*Opium,*" *fays he,* "*Non alia ratione agit in corpus, quam alcohol.*" § "*A fentiment alfo adopted by Huxham, who, fpeaking of the employment of opiates in fmall-pox, fays,* "*They are fimilar in effect to large dofes of fpirituous liquors.*" ‖

*Upon the whole, I truft it has been demonftrated, that the proofs adduced by Dr. Crump in fupport of the ftimulant theory, are both deficient and fallacious; that the explanation it affords of* MANY, *I believe I may truly fay of* ALL *the phenomena in queftion, is unfatisfactory; that facts and experiments inexplicable and inconfiftent therewith do occur* IN GREAT ABUNDANCE: *and having fully fubftantiated thefe charges, the theory in queftion ftands convicted upon the fulleft and faireft evidence,* OF BEING ERRONEOUS; *it will, therefore, only remain to pronounce the fentence which Dr. Crump has paffed upon every erroneous theory, namely,* THAT IT BE CONSIGNED BY ALL TO MERITED OBLIVION. ¶ *And the fooner the fentence is carried into effect, the better will it be for mankind at large; becaufe, independently of avoiding the numerous evils which muft ever be the confequence*

---

* Sydenhami Opera, Sect. 4. Cap. 3.

† Dr. Crump here evidently puts a forced conftruction upon the words of Sydenham, *who does not in this paffage at all allude to its poffeffing ftimulant powers; he merely fpeaks of its cordial properties;* and it would be a reflection upon his fagacity to fuppofe he would have prefcribed a ftimulant medicine with an intention of fupporting the powers of nature when languifhing or oppreffed. It is more probable, I conceive, that he would prefcribe medicines *of a contrary nature, whofe effect would be to diminifh morbid fenfibility and irritability, and take off irregular and painful action. Such a medicine would be the moft fupreme cordial, and fuch is opium. But Dr. Crump ufes the words cordial and ftimulant indifcriminately.*

‡ *Here no particular inftances are pointed out, and the opinion is not agreeable to the general tenor of his writings.*

§ *This paffage by no means proves that Haller was ftruck with its ftimulant properties. All it implies is, that opium acts upon the body in the fame manner as alcohol.* According to this mode of interpretation, an author may be made to fay whatever his Commentator pleafes.

‖ *Dr. Crump here takes the fame liberty with Huxham, which is, at leaft, the third inftance of mifreprefentation that occurs in the fpace of a fingle page.*

¶ "In examining any particular theory, (fays Dr. Crump) our attention will be naturally turned to the proofs adduced in its fupport; the explanation

*consequence of putting erroneous principles into practice, such a measure, if followed by the general adoption of the sedative theory, would lead the way, unless I am greatly deceived, to considerable improvements in the Practice of Surgery as well as medicine.**

*Manchester, Sept.* 6, 1802.

[ To be continued. ]

---

tion it affords of any phenomena in question, and its coincidence with, or repugnance to, particular facts and experiments. *If the first be deficient or fallacious, the next unsatisfactory, and facts and experiments inexplicable or inconsistent occur, the opinions in question are surely to be deemed erroneous,* AND CONSIGNED BY ALL TO MERITED OBLIVION." Inq. p. 144.

* Where any doubt remains, no information ought to be withheld which can tend to elucidate: It is only on this ground, I think it proper to communicate the following intelligence, which comes from very good authority.

" Extract of a Letter from ——— to ———.

" I have been informed by ———, Esq. F. R. S. who went to the East Indies in the Medical line, that the collectors of opium employ thirty men for several months together, during ten hours every day, with their hands and part of their arms constantly immersed in the poppy juice, without experiencing any soporific, much less any poisonous effects from its absorption. Does not such a fact strongly argue against the external application of opium as a remedy? Mr. ——— is a very intelligent and respectable witness."

To which I answer, that it would be establishing a bad precedent to allow a single well authenticated fact on one side of a question, *to invalidate a great number of equally well authenticated facts* on the other side. But there is no necessity for resorting to this plea on the present occasion, for the following reasons.

*If the juice is absorbed,* we cannot be surprized that it should not produce the effects mentioned, *after a few days have elapsed*; (we are not told whether the men are affected by the juice *on their first entering upon the employment*, nor whether they have been previously in the habit of taking opium internally: and yet it is of importance these particulars should be known,) if we recollect that where a person has been accustomed to take opium, either medicinally, or for other purposes, it becomes necessary *to go on increasing the dose progressively*, in order to produce a given effect; (for the same reason, taking tobacco *in any form,* soon ceases to produce effects equally powerful as at first.) *Besides, the exercise requisite for preparing the juice, must operate powerfully in counteracting its effects.*

*If the juice is not absorbed, it proves, either that it renders the lymphatics torpid by its immediately sedative effects upon their coats, or, that friction is necessary to enable them to absorb a sufficient quantity to affect the system at large.*

Some

